# Identification of a Novel Gene, *Osbht*, in Response to High Temperature Tolerance at Booting Stage in Rice

**DOI:** 10.3390/ijms21165862

**Published:** 2020-08-15

**Authors:** Jae-Ryoung Park, Won-Tae Yang, Doh-Hoon Kim, Kyung-Min Kim

**Affiliations:** 1 Division of Plant Biosciences, School of Applied Biosciences, College of Agriculture and Life Science, Kyungpook National University, Daegu 41566, Korea; icd92@naver.com; 2 Coastal Agriculture Research Institute, Kyungpook National University, Daegu 41566, Korea; 3 College of Natural Resources and Life Science, Dong-A University, Busan 49315, Korea; wtyang@dau.ac.kr

**Keywords:** rice, booting stage, QTL, heat stress, abiotic

## Abstract

Rice is one of the world’s leading food crops, and over 90% of the world’s rice production stems from Asia. In particular, an increase of 1 °C in the minimum temperature reduces the quantity of rice by 10%. Therefore, the development of rice varieties that can stably maintain the yield and quality of the rice even under these rapid climate changes is indispensable. In this study, we performed quantitative trait loci (QTL) mapping after treatment with heat stress during the booting stage in rice. We performed a QTL analysis using the Cheongcheong/Nagdong double haploid (CNDH) line and identified 19 QTLs during the 2 year analysis. Of these QTL regions, the 2.2 cM region of RM3709–RM11694 on chromosome 1 was shared among the six traits (heading date; culm length; panicle length; number of tiller; 1000 grain weight; and content of chlorophyll) examined. Rice Microsatellite (RM) 3709–RM11694 contained 27 high-temperature-tolerance candidate genes. Among the candidate genes, *OsBHT* showed a different gene expression level between CNDH75, which is a high-temperature tolerant line, and CNDH11 which is a susceptible line. Although some existing high-temperature-tolerant genes have been reported, *OsBHT* can be used more effectively for the development of heat tolerance in rice.

## 1. Introduction

Rice is one of the world’s major food crops with more than half of the world’s population using it as a staple food. In particular, more than 90% of the world’s rice production (481 million tons) stems from Asia where it is an important crop [1]. However, recently, abnormal climate patterns are occurring worldwide because of rapid climate change. This is causing serious damage to rice production and threatening global food security [2]. According to the World Meteorological Organization, global warming has caused an increase of 1.1 °C in the current global average temperature compared with the pre-industrial period of 1800–1900 and of 0.2 °C compared with the previous 5 years (2011–2015). Temperature has a great effect on the quantity and quality of rice which is in close relationship with the difference in temperature between day and night and solar conditions during the growth period of rice. Suzuki [3] performed a study of rice yield in hot regions and detected a positive effect of high temperature on rice yield regarding the number of tillers and leaf growth in the early stages of rice growth. However, in the late stages of rice growth, carbohydrate accumulation decreases because of increased cell respiration, aging occurs early, and the ripening period is shortened which has a negative effect on yield [4]. In particular, the rice booting and flowering stage are very sensitive to temperature. A high temperature during this growth stage causes significant infertility [5,6]. According to Peng [7], rice yield decreases by 10% whenever the minimum temperature of the rice-growing period increases by 1 °C. In addition, recently, rice production and quality have been severely degraded by summer temperatures in Japan [8]. As such, the rapid rise in temperature caused by climate change is a major cause of rice-yield reduction and quality deterioration. If global warming continues, the damage is expected to become more serious.

As molecular PCR techniques that use DNA markers to amplify DNA and analyze genes are common, gene maps have been extensively generated which allow the analysis of quantitative trait loci (QTL) related to quantitative traits. Tanksley [9] began to use QTL as it became possible to analyze the degree of the genetic effect on the expression of each trait using a quantitative trait locus. QTL mapping is the most accurate method to confirm the relationship between phenotype and genotype [10]. Sen and Churchill [11] also reported on the statistical advantage of QTL mapping. The main agronomic characteristics of rice, such as heading date, culm length, panicle length, and 1000 grain weight, are controlled by multiple loci. The high-temperature tolerance in rice at the booting stage is also a complex property controlled by multiple loci and, thus, is affected not only by genetic factors but also by the environment. The QTL analyses of many aspects of heat tolerance have already been conducted in rice. However, most studies have focused on the flowering stage of the rice-growing stage [12,13]. In recent years, the period of highest temperature worldwide has been lengthening, and the European Climate Change Service, in particular, reported that July of 2019 was the hottest month ever in weather observation. In Asia, where rice production is currently the highest in the world, July is the season of the booting stage in rice. In rice, the flowering and booting stages are the most temperature-sensitive of all developmental stages [14,15]. In particular, when exposed to 40 °C during the flowering stage, the flowering time is delayed and the number of flowers is decreased [16]; moreover, even a slight exposure to an average temperature of 35 °C during flowering causes infertility [14]. Therefore, it can be said that the presence of a high temperature during the booting and flowering stages is a very serious problem in securing rice yield; thus, the mapping of genes involved in high-temperature resistance is essential. In the past, there were many cases in which crop production and economic losses caused by high temperatures were a major economic and social problem [17]. High temperatures also significantly reduce the production of maize and wheat, the world’s major crops [18,19]. As shown here, the world now needs to be prepared for the warming phenomenon.

This research was conducted to perform QTL mapping of high-temperature-resistance genes during the rice booting stage. The results obtained in this study are expected to help develop varieties that are resistant to high-temperature environments due to the fact of global warming and can secure stable quantities in the future.

## 2. Results

### 2.1. Phenotypic Evaluation and Comparison with Agricultural Characteristic

**Figure 1:**
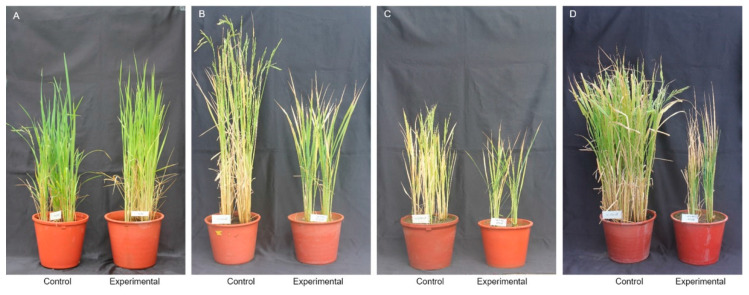
Comparison of phenotypes after high-temperature treatment in rice at the booting stage. The Cheongcheong/Nagdong double haploid (CNDH) 120 line was treated with a high temperature during the booting stage. The phenotype that appeared after the booting stage was assessed. (**A**) Nagdong is a variety that is tolerant to high temperatures; (**B)** Cheongcheong is a variety that is susceptible to high temperatures. Culm length decreased and no heading was observed; (**C**) CNDH11 exhibited a decrease in tiller number after the high-temperature treatment and did not show heading; (**D**) CNDH109 exhibited a great decrease in tiller number after treatment with high temperature.

**Figure 2:**
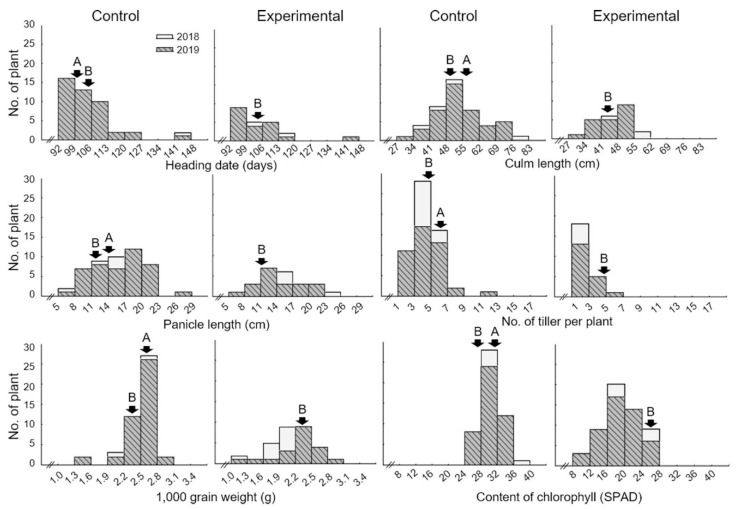
The frequency distribution of heading date, culm length, panicle length, number of tillers, 1000 grain weight, and content of chlorophyll in the CNDH population. As all traits showed a normal distribution, the investigated traits were considered to be quantitative traits. Cheongcheong is a high-temperature resistant variety, and Nagdong is a high-temperature sensitive variety. A, Cheongcheong; B, Nagdong.

Table 1 and Figure 1 show the table and graphs, respectively, that compare the effect of the high-temperature treatment in the control and experimental groups regarding the heading date, culm length, panicle length, number of tillers per plant, 1000 grain weight, and content of chlorophyll in the Cheongcheong, Nagdong, and CNDH 120 lines with the latter being the parental line of the Cheongcheong, Nagdong, and double haploid varieties. There were no significant differences in the effect of the high-temperature treatment between the control and experimental groups regarding the heading date, culm length, and panicle length in all of the Cheongcheong, Nagdong, and CNDH 120 lines. However, the number of tillers per plant, 1000 grain weight, and content of chlorophyll were significantly different (with a probability within 5%) between the experimental and control groups (*p* < 0.05). In addition, the frequency distribution curves of the CNDH line were similar to the normal distribution, and it was confirmed that all of the agriculture characteristics examined here were quantitative traits that were controlled by one or more genes (Figure 2 and Figure S1). The finding that all traits were quantitative traits implies that they are highly influenced by the environment [20]. Cheongcheong is a high-temperature susceptible variety. So, heading, panicle length, culm length, and number of tiller per plant, and 1000 grain weight could not be measured. Therefore, no data are displayed in Table 1.

### 2.2. Analysis of QTLs Associated with Heat Tolerance

A total of 788 SSR markers were used for genetic mapping using the Cheongcheong/Nagdong DH line, and a total of 423 SSR markers showed polymorphism in the polymorphism test performed for the Cheongcheong and Nagdong lines. Among the 423 SSR markers selected through the polymorphism assay, codominance was observed; thus, 143 SSR markers that amplified all of the parental regions were selected and used for gene mapping. The average distance between markers of the linkage map of the Cheongcheong/Nagdong DH line was 10.6 cM. The number of markers per chromosome was 19–50, and the markers were distributed evenly on each chromosome (Figure S2). After the administration of the high-temperature treatment to the CNDH line, the QTL analysis was performed using the composite interval mapping (CIM) method of Windows QTL Cartographer 2.5 based on phenotypic data and genotyping information. The QTL analysis of high-temperature tolerance in rice led to the detection of 16 QTLs on chromosome 1 and three QTLs on chromosome 12. The loci corresponding to heading date (qHdd1 and qHdd1-2), culm length (qCl11 and qCl11-2), panicle length (qPl1, qPl1-2, and qPl12), number of tillers per plant (qNt1), 1000 grain weight (qTgw1, qTgw1-1, and qTgw12), and content of chlorophyll (qCc1 and qCc1-3) that had an logarithm of the odds (LOD) value of 3.0 or higher QTL was explored (Table 2 and Figure S2). The heading date of 2018 was searched in RM212–RM1297 and RM11605–RM3530 on chromosome 1, the LOD values of which were 4.15 and 2.70. The heading date of 2019 was detected in RM212–RM1297 on chromosome 1, with an LOD value of 4.20. The QTLs related to the high-temperature tolerance regarding heading date over the 2 year period had an LOD value of 3.0 or higher in RM212–RM1297. Culm length in 2018 was detected in RM212–RM3411 and RM11605–RM3530 on chromosome 1 and in RM8216–RM28816 on chromosome 12 with LOD values of 4.58, 2.70, and 2.95, respectively. Culm length in 2019 was detected in RM212–RM1297 on chromosome 1, with an LOD value of 4.39. The QTLs associated with the high-temperature tolerance regarding culm length over the 2 year period had an LOD value of 3.0 or higher in RM212–RM3411. The panicle length of 2018 was searched in RM212–RM3411 and RM1297–RM3530 on chromosome 1, yielding LOD values of 4.24 and 2.70, respectively. The panicle length of 2019 was detected in RM212–RM1297 on chromosome 1 and RM8216–RM1159 on chromosome 12, with LOD values of 3.97 and 3.12, respectively. The QTLs related to high-temperature tolerance over a 2 year period were detected to have an LOD value of 3.0 or higher in RM212–RM3411. The number of tillers per plant in 2018 was detected in RM212–RM1297 on chromosome 1 with an LOD value of 3.45. In 2018, the 1000 grain weight was detected in RM212–RM11694 on chromosome 1 and in RM8216–RM1159 on chromosome 12, with LOD values of 3.80 and 3.23, respectively. The 1000 grain weight for 2019 was detected in RM212–RM11669 on chromosome 1 with an LOD value of 3.78. The QTLs associated with high-temperature tolerance during the 2 year period regarding the 1000 grain weight had an LOD value of 3.0 or higher in RM212–RM1297 on chromosome 1. The content of chlorophyll in 2018 was detected in RM3790–RM11669 and RM11605–RM3530 on chromosome 1 with LOD values of 3.09 and 2.98, respectively. The content of chlorophyll for 2019 was detected in RM3709–RM11669 and RM11605–RM3530 on chromosome 1 with LOD values of 2.98 and 3.13, respectively. The QTLs related to high-temperature tolerance of chlorophyll content for the 2 year period had an LOD value higher than 3.0 in RM3709–RM11669. Finally, the search for high-temperature-tolerance QTLs regarding heading date, culm length, panicle length, number of tillers per plant, 1000 grain weight, and content of chlorophyll over the 2 year period revealed that RM3709–RM11694 on chromosome 1 had an LOD value of 3.0 or higher.

### 2.3. Search for Candidate Genes Associated with Heat Tolerance Based on QTL Mapping 

The analysis of high-temperature-tolerance QTLs for heading date, culm length, panicle length, number of tillers per plant, 1000 grain weight, and content of chlorophyll detected 16 QTLs on chromosome 1. These QTLs were all located in the range of RM212–RM3530 on chromosome 1. However, RM3709–RM11694 (which spanned a region of 2.2 cM) was the region shared by all agricultural traits. These SSR markers were analyzed in NCBI which led to the detection of 27 open reading frames (ORFs) (Table S2), all of which were associated with heat tolerance. The ORFs are classified in Supplementary Materials Table S2 according to function [21]. Rice Microsatellite (RM) 3709–RM11694 (2.2 cM) contains candidate genes related to cell structure, plant hormone regulation, plant defense, protein kinase, heat shock protein, and signaling. The candidate genes for cell structure included membrane biosynthesis, lipid metabolism regulation, pore structure regulation, and cell–wall structural proteins. The candidate genes for plant hormone regulation included the ABA, GA, and Auxin regulatory genes. The candidate genes for plant defense were the WRKY transcription factor, glycosyl transferase, prenyltransferase domain-containing protein, RING-type domain-containing protein, potassium channel, and an ethylene-responsive transcription factor. It also included keyase (a serine/threonine kinase) and the protein kinase C substrate keyase, a phosphorylation reaction catalyst. In addition, two genes that respond to heat-shock (i.e., a heat-shock factor and HSP20-like chaperone domain-containing protein) and two genes that are involved in signaling (i.e., the Ras-related protein RIC2 and a GTP-binding protein were also included.

### 2.4. Candidate Genes Expression Level at Booting Stage with Treatment of High Temperature

**Figure 3:**
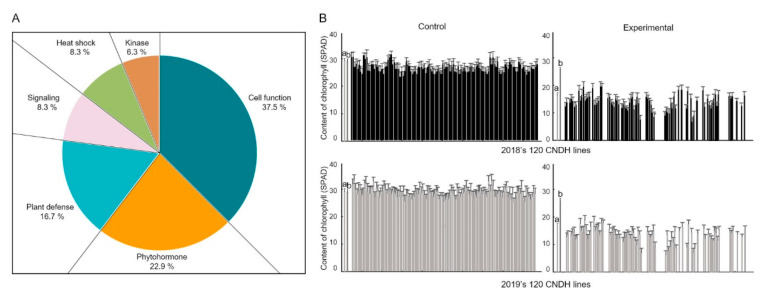
Candidate genes between RM212 and RM3411 and changes in chlorophyll content during high-temperature treatment. (**A**) Forty-eight candidate genes were distributed by function. Among the candidate genes, the gene responsible for cell function was detected most frequently, and heat shock protein accounted for 8.3%. (**B**) Changes in chlorophyll content when high temperature was the treatment in rice at the booting stage. a, Cheongcheong; b, Nagdong.

**Figure 4:**
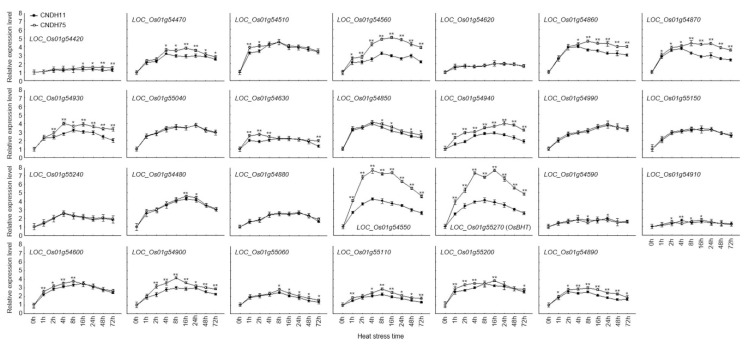
Analysis of relative expression levels of candidate genes in high-temperature susceptible and tolerance lines. Expression analysis of candidate genes in rice at the booting stage exposed to 42 °C heat stress in a growth chamber, with rice leaf sampled at different time points at 0 h, 1 h, 2 h, 4 h, 8 h, 12 h, 16 h, 24 h, 28 h, and 72 h. Data are presented as the mean ± SD (*n* = 3), Student’s *t*-test. CNDH11 is the high-temperature susceptible line, and CNDH75 is the high-temperature tolerance line. * Significant at the 0.05 level; ** significant at the 0.01 level.

According to the rice genome annotation project database Rapdb [22] and RiceXpro [23], twenty-seven candidate genes are located in the target region. Of these, 37.5% are associated with cell function, 22.9% with hormones, 16.7% with plant defense, 8.3% with signaling, 8.3% with heat shock protein, and 6.3% with kinase (Figure 3A,B). To compare the expression levels of candidate genes during high-temperature stress, we used a quantitative real-time analysis of 27 candidate high-temperature resistance genes, CNDH75, which is from the high-temperature tolerance CNDH line, and CNDH11, which is from the high-temperature susceptible CNDH line (Figure 4). Eight of the 27 candidate genes showed differences between CNDH11 and CNDH75. *LOC_Os01g54560, LOC_Os01g54870, LOC_Os01g54940, LOC_Os01g54550*, and *LOC_Os01g55270* showed higher expression levels in CNDH75 at 1 h, 2 h, 4 h, 8 h, 16 h, 24 h, 48 h, and 72 h than CNDH11; *LOC_Os01g54930* showed higher expression levels in CNDH75 at 2 h, 4 h, 8 h, 16 h, 24 h, 48 h, and 72 h than CNDH11; *LOC_Os01g54470, LOC_Os01g54860* CNDH75 showed higher expression levels at 4 h, 8 h, 16 h, 24 h, 48 h, and 72 h than CNDH11. Among these genes, *LOC_Os01g54550* and *LOC_Os01g55270* (*OsBHT*, *Oryza sativa* booting stage high-temperature tolerance), which belong to the heat shock protein group, showed a significantly difference expression level between CNDH11 and CNDH75 than other genes.

### 2.5. Analysis of Phylogenetic Tree and Homology Sequence

**Figure 5:**
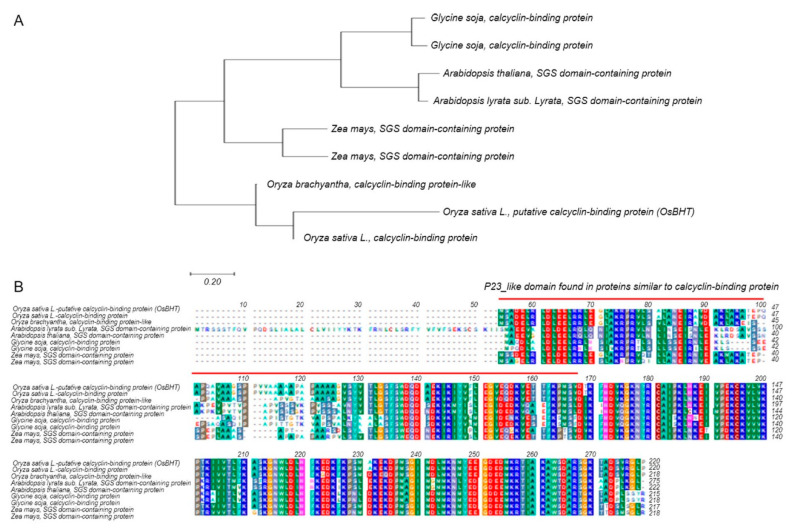
Sequence analysis of *Oryza sativa* Booting stage Heat Tolerance (*OsBHT*). (**A**) Analysis of the relationship between the *OsBHT* gene and homology gene by phylogenetic tree. The phylogenetic tree was constructed by the parsimony method with 1000 bootstrap replicates. (**B**) As a result of comparing the protein sequences of the homologous genes of *OsBHT*, a very high similarity was shown in *Oryza sativa*, *Arabidopsis thaliana*, *Zea mays*, and *Glycine soja*.

**Figure 6:**
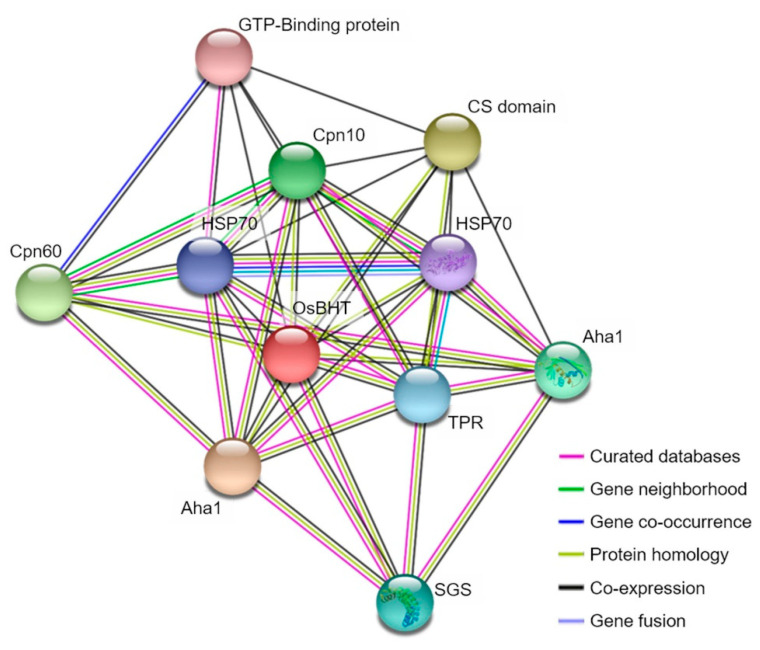
*OsBHT* protein interaction prediction. *OsBHT* interact with the heat shock protein. Signaled by the guanosine triphosphate (GTP)-binding protein when plants are exposed to high temperatures, Hsp70 and Hsp90 work together with multiple co-chaperones to prevent protein denaturation.

The chlorophyll content showed the largest difference between the experimental group and the control group at the time of rice high-temperature treatment at the booting stage, and the chlorophyll content decreased with a significant probability of 1% at the high-temperature treatment (Figure 3). The chlorophyll content of the sensitive lines to high temperature was greatly reduced, but the chlorophyll content of the maternal, Nagdong, was not significantly reduced even at high temperatures. Therefore, we have found candidate genes that are associated with chlorophyll content. *Oryza sativa* Booting stage Heat Tolerance (*OsBHT*) encodes calcyclin-binding protein among candidate genes. Calcyclin-binding protein is one of the Hsp90 co-chaperones, a kind of heat shock protein. The BLAST results revealed a very high similarity of amino acid to calcyclin-binding protein of *Oryza brachyantha* (85% identity and 88% similarity). *OsBHT* belongs to the Hsps-p23-like calcyclin-binding protein superfamily. The sequences were very similar to those of *Zea mays* SGS domain-containing protein, *Arabidopsis thaliana* SGS domain-containing protein, and *Glycine soja* calcyclin-binding protein (Figure 5). We also predicted functional partners using the domain of *OsBHT*. *OsBHT* interacts with eight proteins: GTP binding protein, CS domain, Cpn10, Cpn60, Hsp70, Aha1, TPR domain, and SGS domain (Figure 6).

## 3. Discussion

High temperatures cause a variety of injuries in plants. In particular, rice exhibits damage such as deterioration in quality and quantity [24,25]. Therefore, many studies have been performed to develop high-temperature-tolerant varieties using traditional breeding and molecular biological methods in rice which is the world’s leading food crop [12,26,27]. In particular, research is currently under way to localize high-temperature-tolerance genes using QTL; however, most of them are concentrated in the flowering stage of the rice-growing stage [13,28,29]. In this research, heat tolerance QTL mapping was conducted in July and August when the temperature rises most during the year. July and August correspond to the season of the rice booting stage. After planting, the initial growth of the rice was performed under normal conditions; upon reaching the booting stage, the rice was divided into an experimental group and a control group and subjected to high-temperature treatment. The rice was re-grown under normal conditions after the end of the booting stage, and the major agricultural traits of rice were examined such as heading date, culm length, panicle length, number of tillers per plant, 1000 grain weight, and content of chlorophyll. There was no significant difference between the experimental group and the control group during the high-temperature treatment of rice regarding the booting stage, heading date, culm length, and panicle length. However, the number of tillers per plant, 1000 grain weight, and content of chlorophyll were significantly different after the high-temperature treatment in rice (*p* < 0.05). Because of the high temperature of the rice environment in the booting stage, the number of tillers per plant, 1000 grain weight, and content of chlorophyll were decreased compared with the control group.

After the treatment of rice with high-temperature during the booting stage, the QTL mapping analysis using the major rice traits, such as heading date, culm length, panicle length, number of tillers per plant, 1000 grain weight, and content of chlorophyll resulted in the identification of 13 QTLs on chromosome 1 and two QTLs on chromosome 12 with an LOD value of 3.0 or higher. Among these QTLs, the 2.2 cM segment corresponding to RM3709–RM11694 on chromosome 1 was the QTL region that was commonly found in all investigated traits after rice was subjected to high-temperature treatment. In the linkage map, the distribution of markers per chromosome was uniform and the density was relatively high; thus, it can be considered that the main QTL was not lost. The 2.2 cM of RM3709–RM11694 on chromosome 1, which was identified through QTL mapping, contained 27 ORFs which corresponded to various proteins involved in cell function, hormone, plant defense, kinase, heat shock, and signaling (Table S1). In this study, high-temperature treatment in rice at the booting stage yielded significant differences in the number of tillers per plant, 1000 grain weight, and content of chlorophyll compared with the control group (*p* < 0.05) [30]. Among these functions, we focused on finding genes related to these traits. As a result, we focused on *OsBHT*. *OsBHT* is an Hsps-p23-like calcyclin-binding protein that is a type of heat shock protein. Guo et al. [31] overexpressed *OsHSP20* in rice and developed a line that is tolerant to high temperatures and does not exhibit a reduction in chloroplast content. *OsBHT* belongs to Hsps-p23-like superfamily. To date, about 10 Hsp90 co-chaperones have been identified. Protein P23, one of the co-chaperones, functions to suppress ATPase activity by binding to Hsp90 and suppressing the structural change of Hsp90. In particular Xu et al. [32] reported that overexpression of the Hsp90 gene prevents loss and decrease of chlorophyll. In the present study, the chlorophyll content was reduced with a significant probability when rice was treated at the high temperature during the booting stage, and *OsBHT* related to heat shock protein was detected among the candidate genes in the QTL mapping results. In addition, *OsBHT* is an HSP-like chaperone-domain-containing protein which is an Hsp90 co-chaperone. Therefore, the results of this study can show a high level of reliability. In addition, we confirmed the sequence homology with *OsBHT* from rice, *A. thaliana*, *Z. mays*, and *G. soja*, and showed high similarity to calcyclin-binding protein, a kind of Hsp90-binding protein. All of these proteins are known to be eukaryotic stress-resistant genes and useful in heat shock conditions [33,34,35]. *OsBHT* interacts with GTP-binding domain, Cpn10, Cpn60, Hsp70, Aha1, TPR domain, and SGS domain. The main function of the GTP-binding protein is to make a signal transduction link between various receptors and to transmit signals from the cell membrane through the receptors [36]. It plays a role of repeated cycling of GTP-binding domain activity and inactivity which is performed by GTPase activity and GTP-binding force. Cpn60 helps with protein folding by hydrolysis of ATP. However, Cpn10 suppresses the activity of GTPase [37]. In a normal environment, Hsp70 is involved in the folding of newly synthesized proteins for ATP-dependent molecular chaperone, and it has been clarified that it has a function of collecting multiple proteins and binding to a protein complex [38]; under stress conditions, it is known to prevent unfolding and denaturation of cellular proteins due to the fact of stress and to maintain the normal function of cells [39]. Hsp90 also has a similar function to Hsp70 [40]. Hsp90, which exists in various forms, is the most abundant cellular chaperone and is responsible for the ATP-dependent refolding function of denatured and unfolded proteins [41]. Thus, it functions as part of the cell’s defense against stress. When cells are exposed to heat or other environmental stresses, aggregation of unfolded proteins is prevented by pathways that catalyze their refolding or degradation [42]. The Hsp90 protein forms a dimer, and its function is regulated according to a periodic morphological change due to the ATP binding and degradation. Hsp90 regulates the activity of Hsp90 by functions such as multiple co-chaperone regulating the ATPase activity of Hsp90 and mediating the binding between Hsp90 and client protein [43]. To date, many types of Hsp90 co-chaperones have been identified. Many *OsBHT* discovered in this study also interacts with co-chaperone. Aha1 is the only co-chaperone that promotes the ATPase activity of Hsp90 [44]. In plants, a protein with a structure similar to that of the R protein, which is an immunosensor that recognizes external antigens, exists and is classified as an NLR (Nod-like receptor) [45]. At this time, Hsp90 co-chaperone Sgt1 plays an important role in stabilizing or activating NLR proteins [46]. Sgt1 is composed of TPR, CS (CHORD-containing proteins and Sgt1), and SGS (Sgt1-specific) domains [47]. The SGS domain of Sgt1 acts as a mediator that induces the NLR protein into Hsp90.

In addition, various candidate genes related to heat tolerance were discovered in this region, RM212–RM3411 on chromosome 1. Plant defense genes, such as the genes encoding the WRKY transcription factor [48], protein in the glycosyl transferase family [49], prenyltransferase domain-containing protein [50], similar to potassium channel [51], similar to ethylene-responsive transcription factor [52], and RING-type domain-containing protein [53], which confer tolerance to various abiotic stresses, such as heat, salt, and drought, were found as candidate genes in the QTL region (RM212–RM3411 on chromosome 1). Plants also have environment-specific genes to deal with multiple environmental stresses; however, most abiotic stress genes or plant defense genes share pathways for resistance to multiple environmental stresses [54]. In addition, various plant hormones act as signaling factors in various stressful environments and resist these stresses [55]. Plant hormones are also shared among the various pathways that deal with stressful environments, rather than have stress-specific expression [56]. In addition to plant hormones, this study also detected candidate genes such as those similar to cellulose synthase and glycosyl transferase, which are involved in the high-temperature tolerance of rice in the booting stage and confer tolerance to high-temperature and various biotic and abiotic stresses by modifying the structure of cells [32,57].

Here, we conducted QTL mapping for the high-temperature tolerance of rice in the booting stage and identified various candidate genes in a region of 2.2 cM corresponding to RM3709–RM11694 on chromosome 1. Moreover, proteins with domains similar to the gene families of these candidate genes have already been used for developing high-temperature-tolerant varieties [58]. Takeuchi et al. [59] performed cold-tolerance QTL mapping during the rice booting stage and identified candidates in a position similar to RM3709–RM11694 on chromosome 1. In addition, Zhao et al. [60] conducted heat tolerance QTL mapping in rice and found candidates that also mapped at similar locations. However, Ye et al. [31] explored QTLs on chromosomes 1 and 4. The slight differences in the results of QTL mapping obtained in these studies can be explained by the differences in the genetic and environmental properties of the materials used in the experiments [61]. The high-temperature-tolerance QTL of rice at the booting stage detected in this study was derived from the *japonica* type, Nagdong rice, and is expected to be useful for the development of a Japonica heat-tolerant variety.

## 4. Materials and Methods 

### 4.1. Plant Material and Field Design

In this study, a double haploid line (CNDH) that was obtained through a crossing of the Cheongcheong (*O. sativa* L. ssp. *indica*) and Nagdong (*O. sativa* L. ssp. *japonica*) varieties of rice was used as experimental material for the QLT analysis of rice high-temperature tolerance at the booting stage. CNDH 120 lines were developed by another culture of the F_1_ generation derived from a crossing between Cheongcheong and Nagdong. Among the CNDH lines, 120 lines that did not exhibit segregation but possessed a uniform agronomic character were selected and used as experimental materials for this research. Seeds were sterilized using a disinfectant and incubated at 33 °C for 3 days. On 21 April, 2018 and 23 April, 2019, they were seeded at Kyungpook National University’s experimental farm in Gunwi, South Korea, respectively. The amount of fertilization was N-P2O5-K2O = 9–4.5–5.7 kg/10 a, and rice was cultivated according to the Agricultural Science and Technology Research Standards of the Rural Development Administration. Plants were transferred to the growth chamber at the booting stage and were grown under high-temperature conditions (Table S2) in the growth chamber during the booting stage. When the booting stage was over and the flowering stage began, the plants grew again under normal conditions.

### 4.2. Phenotypic Evaluation

On 15 September, 2018 and 17 September, 2019, the major agricultural traits of rice (i.e., heading date, culm length, panicle length, number of tillers per plant, 1000 grain weight, and content of chlorophyll) were investigated. The heading date was calculated as the time from sowing to flowering. The culm length was measured from the donation of rice paddies to the neck of the panicle of rice, and panicle length was measured from the neck of the panicle to the grain of rice. The number of tillers per plant were a measure of the effective tillering. Moreover, 1000 grain weight corresponded to the weight of 1000 seeds, and the content of chlorophyll was measured using a portable chlorophyll meter (SPAD-502, Minolta Camera Co. Ltd., Osaka, Japan). The recordings of the characteristics of all of the agricultural traits investigated in this research were repeated in 10 plants each, and statistical analysis was performed using the SPSS program (IMMSPSS Statistics, version 22, NC) [62].

### 4.3. Construction of a Genetic Map and QTL Analysis of Heat Tolerance in Rice

To analyze the QTLs related to rice high-temperature-tolerance genes, Windows QTL Cartographer 2.5 was used. In addition, a genetic map with an average distance of 10.6 cM was created using Mapmaker version 3.0 with 222 SSR markers. For the CNDH 120 line, the values of heading date, culm length, panicle length, number of tillers per plant, 1000 grain weight, and content of chlorophyll were analyzed in the Kosambi Function using the Composite Interval Mapping (CIM) method and a threshold LOD value of 3.0 or higher. The naming of the QTL was performed according to the method proposed by McCouch [63].

### 4.4. Gene Information Analysis

The QTL mapping alone cannot satisfy the use and satisfaction in actual breeding. Therefore, the analysis and discovery of the candidate gene group is an important factor in QTL analysis. The discovery of candidate genes can overcome the deficiencies of QTL analysis. Rapdb [22] and RiceXpro [23] can create physical maps and annotate candidate genes. The open reading frame (ORF) found among the SSR markers was classified and functionally analyzed, and the heat tolerance was filtered. The sequence analysis and protein prediction were analyzed using DNASIS for Windows (Hitachi Software Engineering) and ExPASy [64]. The homology multiple sequence comparison was conducted by NCBI [65] and BioEdit 7.0 [66].

### 4.5. Analysis of Candidate Genes Expression Level

Among the CNDH lines, CNDH75, the high-temperature tolerance line, and CNDH11, the high-temperature susceptible line, were treated at a high temperature of 42 °C in the growth chamber at the rice booting stage. Rice leaves were sampled at 0 h, 1 h, 2 h, 4 h, 8 h, 16 h, 24 h, 48 h, and 72 h while being subjected to high-temperature treatment during the rice booting stage. The RNeasy plant mini kit (QIAGEN, Hilden, Germany) was used to extract total RNA from rice leaves, and 1 μg of RNA was used as a template for cDNA synthesis using oligo-dt primer and transcriptase (Invitrogen, Carlsbad, California, USA). The qRCRBIO cDNA Synthesis kit (Cat No. PB30.11-10, PCRBIOSYSTEM, Wayne, Pennsylvania, USA) was used for cDNA synthesis. Quantitative real-time PCR was performed using the Eco Real-Time PCR System. For the reaction solution for quantitative real-time PCR, 2× qRCRBIO SyGreen Blue Mix 10 μL, cDNA 1 μL, forward primer 1 μL (10 pmol/μL), reverse primer 1 μL (10 pmol/μL), and ddH2O were used to make a final volume of 20 μL. OsActin was used as a control group, and each reaction was run in triplicate to calculate the mean and standard deviation.

### 4.6. RNA Extraction and PCR Protocol

The RNA from the Chungcheong, Nagdong, and double haploid lines was extracted using an RNeasy plant mini kit (QIAGEN, Hilden, Germany). Fresh rice leaves were ground with liquid nitrogen, and 100 mg of rice-leaf powder was used in experiments. Subsequently, 450 μL of RLT buffer was added to the powder and mixed using a vortex. All solutions were added to the QIAshredder spin column and centrifuged at 13,000 rpm for 2 min. Ethanol (100%) was added to 0.5 volumes of the solution that was filtered through the column and mixed. The mixed solution was placed in an RNeasy mini column and centrifuged at 13,000 rpm for 15 s. Thereafter, the column was washed with 700 μL of RW1 buffer and 500 μL of RPE buffer and centrifuged at 13,000 rpm for 2 min without adding any other reagents to dry the column completely. Finally, it was dissolved in 30 μL of Rnase-free water. The RNA was diluted to a final concentration of 80 ng/μL using Rnase-free water and was then used for cDNA synthesis. The concentration and quality of the obtained RNA were estimated using an ultra-microspectrophotometer (ND-2000; Nanodrop, Waltham, Massachusetts, USA). For cDNA synthesis, the qPCRBIO cDNA Synthesis Kit (PCRBIOSYSTEMS, USA) was used: 4 μL of 5× cDNA synthesis mix, 1 μL of 20× RTase, and 80 ng of RNA were added and the final volume was adjusted to 20 μL, followed by reaction at 42 °C for 30 min. The synthesized cDNA (80 ng) was used for PCR. The PCR mixture included 80 ng of cDNA, 10 pmol of forward and reverse primers, 2.5 mM dNTP mixture, 1.0 U of Ex Taq polymerase (RR001A, TaKaRa, Seoul, Korea), 2.5 μL of 10× Ex buffer (50 mM KCl, 20 mM Tris-HCl, pH 8.0, and 2.0 mM MgCl_2_), and nuclease-free water (Qiagen, Cat. No. 129114), to adjust the total volume to 30 μL. The protocol of the PCR (C1000, BioRad, Hercules, California, USA) amplification was as follows: pre-denaturation at 94 °C for 5 min; followed by 35 cycles of denaturation at 94 °C for 30 s, annealing at 55 °C for 30 s, and extension for 35 min at 72 °C; and a final extension of 5 min at 72 °C. In further analyses, the PCR product was sequenced by SOLGENT (Daejeon, Korea). Sequencing analysis was used for homology searches using the BLAST program at the NCBI [65] database.

## 5. Conclusions

The QTL mapping was carried out to search for genes that are tolerant to high temperature at the booting stage in rice. When the major agronomic traits after high temperature treatment of the CNDH line were evaluated, they were almost negatively affected. In addition, these major agronomic traits showed a normal distribution while showing a continuous distribution. It means that high-temperature tolerance is a quantitative trait which has an expression that is regulated by one or more genes. Using the data of main agricultural traits phenotypes after high-temperature treatment for 2 years, QTL mapping results were commonly mapped to RM3709–RM11694 on chromosome 1. Rice microsatellite (RM) 3709–RM11694 contained 27 candidate genes related to cell function, signaling, hormone, kinase, and heat shock protein for high-temperature tolerance. Using these candidate genes, we checked the relative gene expression levels in susceptible and tolerance line after high temperature treatment. Of these, *OsBHT*, which belongs to heat shock protein, showed a significant difference between high-temperature susceptible and tolerance lines after high-temperature treatment. *OsBHT* kept a high expression level in the high-temperature resistant line. *OsBHT* was very similar when comparing homology with genes present in *A. thaliana*, *G. soja*, *O. sativa*, and *Z. mays*. In an environment where high temperatures frequently occur due to the fact of abnormal weather, the high-temperature resistance gene *OsBHT*, newly added through this study, will be useful for the development of new rice varieties that can secure a stable water amount even at high temperatures.

## Figures and Tables

**Table 1:** Plant phenotypes of 120 Cheongcheong/Nagdong double haploid (CNDH) lines from a cross of the Cheongcheong and Nagdong varieties.

Plant Traits	Year	Parents						DH Population		
		Cheongcheong			Nagdong					
		Control	Experimental	*p*-Value	Control	Experimental	*p*-Value	Control	Experimental	*p*-Value
Heading date (Days)	2018	101.7 ± 1.2 ^z^	-	-	104.5 ± 1.5	105.2 ± 1.3	0.054	103.7 ± 10.1	104.7 ± 6.7	0.854
	2019	102.6 ± 0.8	-	-	105.3 ± 1.1	106.3 ± 1.2	0.059	104.0 ± 8.3	105.4 ± 6.3	0.633
Culm length (cm)	2018	55.8 ± 1.6	-	-	53.7 ± 1.4	47.8 ± 2.4	0.094	53.9 ± 10.2	46.7 ± 7.6	0.007 **
	2019	53.7 ± 2.4	-	-	52.8 ± 1.8	48.1 ± 2.2	0.005	54.4 ±10.2	46.4 ± 7.2	0.003 **
Panicle length (cm)	2018	14.7 ± 2.8	-	-	12.6 ± 3.4	11.2 ± 2.5	0.080	15.6 ± 4.5	15.6 ± 4.1	0.947
	2019	12.6 ± 1.7	-	-	11.9 ± 1.2	10.6 ± 2.5	0.150	16.0 ±4.7	14.9 ± 4.2	0.339
Number of tillers	2018	6.7 ± 1.5	-	-	5.4 ± 1.7	5.0 ± 1.1	0.388	5.0 ± 1.1	2.5 ± 1.1	<0.001 ***
	2019	6.2 ± 1.2	-	-	5.1 ± 0.7	4.9 ± 0.9	0.588	4.7 ± 2.1	2.4 ± 1.5	<0.001 ***
1000 grain weight (g)	2018	2.6 ± 0.1	-	-	2.4 ± 0.3	2.3 ± 0.2	0.151	2.6 ± 0.3	1.9 ± 0.3	0.031 *
	2019	2.5 ± 1.3	-	-	2.3 ± 0.1	2.2 ± 0.1	0.003 **	2.6 ±0.3	1.8 ± 0.2	0.017 *
Contents of chlorophyll (SPAD)	2018	29.5 ± 2.6	18.7 ± 2.7	<0.001 ***	28.9 ± 1.2	26.7 ± 0.9	<0.001 ***	30.3 ± 2.8	19.6 ± 4.5	<0.001 ***
	2019	28.8 ± 1.7	17.5 ± 2.8	<0.001 ***	27.8 ± 0.9	25.7 ± 1.2	0.002 **	30.2 ±2.6	18.6 ± 4.3	<0.001 ***

^Z^ The data are presented as the mean ± standard deviation; * significant at the 0.05 level; ** significant at the 0.01 level; *** significant at the 0.001 level.

**Table 2:** QTL related to the agronomic characters of the Cheongcheong/Nagdong DH population.

Characteristics	Year	QTL	Chromosome	Interval Markers ^z^	LOD	Additive effect ^y^	*r* ^2 x^	Increasing Effects ^w^
Heading date	2018	qHdd1	1	RM212–RM1297	4.15	−15.0	0.2	Nagdong
		qHdd1-1	1	RM11605–RM3530	2.70	−16.1	0.2	Nagdong
	2019	qHdd1-2	1	RM212–RM1297	4.20	−11.2	0.2	Nagdong
Culm length	2018	qCl1	1	RM212–RM3411	4.58	−7.2	0.2	Nagdong
		qCl1-1	1	RM11605–RM3530	2.70	−5.7	0.2	Nagdong
		qCl112	12	RM8216–RM28816	2.95	−6.3	0.2	Nagdong
	2019	qCl1-2	1	RM212–RM1297	4.39	−7.7	0.2	Nagdong
Panicle length	2018	qPl1	1	RM212–RM3411	4.24	−2.2	0.3	Nagdong
		qPl1-1	1	RM1297–RM3530	2.70	−2.1	0.3	Nagdong
	2019	qPl1-2	1	RM212–RM1297	3.97	−2.2	0.2	Nagdong
		qPl112	12	RM8216–RM1159	3.12	−2.1	0.2	Nagdong
Number of tillers	2018	qNt1	1	RM212–RM1297	3.45	−0.5	0.2	Nagdong
1000 grain weight	2018	qTgw1	1	RM212–RM11694	3.80	−0.3	0.2	Nagdong
		qTgw 12	12	RM8216–RM1159	3.23	−1.2	0.1	Nagdong
	2019	qTgw1-1	1	RM212–RM11669	3.78	−0.3	0.2	Nagdong
Content of chlorophyll	2018	qCc1	1	RM3709–RM11669	3.09	−3.3	0.2	Nagdong
		qCc1-1	1	RM11605–RM3530	2.98	−4.6	0.3	Nagdong
	2019	qCc1-2	1	RM3709-RM11669	2.98	−3.4	0.2	Nagdong
		qCc1-3	1	RM11605–RM3530	3.13	−4.4	0.3	Nagdong

Hdd, heading date; Cl, culm length; Pl, panicle length; Nt, number of tillers; Tgw, 1000 grain weight; Cc, content of chlorophyll. ^z^ Interval markers are those within the significance threshold on each border of the QTL range. ^y^ The proportion of evaluated phenotype variations attributable to a particular QTL was estimated using the coefficient of determination (*r*^2^). ^x^ Positive values of the additive effect indicate that alleles from Samgang are in the direction of increases in the traits. ^w^ Increased allele is the source of the allele causing an increase in the measured trait.

## Supplementary Materials

Supplementary materials can be found at https://www.mdpi.com/1422-0067/21/16/5862/s1, Figure S1: Comparison of agricultural traits of control and experimental groups when the rice in the booting stage was treated to high temperature. Figure S2: The chromosomal location of QTLs associated with heat tolerance in CNDH population. Table S1: Twenty-seven candidate genes identified between the RM212 and RM3411 markers and their ORFs which include various proteins related to heat tolerance. Table S2: Environmental settings for high-temperature treatment in the indoor growth chamber.
