# Supplementary material for: Identification of a Novel Gene, Osbht, in Response to High Temperature Tolerance at Booting Stage in Rice
Source: Int J Mol Sci. 2020 Aug 15;21(16):5862. doi: 10.3390/ijms21165862 (PMC7461545; doi:10.3390/ijms21165862)
Supplement: Supplementary file 1 [file ijms-21-05862-s001.pdf]

Table S1. Twenty-seven candidate genes identified between the RM212 and RM3411 markers and their ORFs, which include various proteins related to heat tolerance.

Marker interval	Function	Locus	Description	No. of genes
RM212-RM1297	Cell function	Os01g0748000	A protein that fuses vesicles to the cytoplasmic membrane	1
		Os01g0748500	Lipolytic enzyme, G-D-S-L family protein	1
		Os01g0748900	Membrane attack complex C9 family protein	2
		Os01g0749400	HAD-superfamily hydrolase subfamily IIB protein	3
		Os01g0750300	Similar to Cellulose synthase	3
		Os01g0752200	Enoyl-CoA hydratase/isomerase domain-containing protein	1
		Os01g0752300	Similar to 60S ribosomal protein L18a-1	1
		Os01g0753000	Colicin E3, catalytic domain-containing protein	1
		Os01g0754200	Glycosyl transferase, family 48 protein	5
	Hormone	Os01g0750400	Leucine-rich repeat, plant-specific containing protein	1
		Os01g0752100	Cyclin-like F-box domain-containing protein	1
		Os01g0753100	Alcohol dehydrogenase superfamily, zinc-containing protein	2
		Os01g0753500	Transcriptional factor B3 family protein	1

	Os01g0756200	Similar to VirE2-interacting protein VIP1	1
	Os01g0757200	Similar to GA 2-oxidase 4	5
Plant defense	Os01g0750100	Similar to WRKY transcription factor	3
	Os01g0752600	Glycosyl transferase, family 19 protein	1
	Os01g0754600	Prenyltransferase domain-containing protein	1
	Os01g0755700	RING-type domain-containing protein	1
	Os01g0756700	Similar to Potassium channel	1
	Os01g0752500	Similar to Ethylene-responsive transcription factor 6	1
Kinase	Os01g0748600	Protein kinase domain-containing protein	2
	Os01g0752400	Similar to Glucosidase II beta subunit precursor	1
Heat-shock	Os01g0749300	Similar to Heat-shock factor	2
	Os01g0757500	HSP20-like chaperone-domain-containing protein	2
Signaling	Os01g0750000	Similar to Ras-related protein RIC2	3
	Os01g0752700	Similar to GTP-binding protein	1

Table S2. Environmental settings for high temperature treatment in the indoor growth chamber.

Step	Time	Duration (min)	Temperature (°C)	RH(%)	Light (μmol $m^{-2}s^{-1}$)
1	06:30-07:00	30	27	75	330
2	07:00-07:30	30	30	75	460
3	07:30-08:00	30	35	70	580
4	08:00-08:30	30	38	70	580
5	08:30-14:30	360	42	70	580
6	14:30-15:30	60	35	70	580
7	15:30-16:30	60	30	70	580
8	16:30-17:30	60	27	75	460
9	17:30-18:30	60	24	75	330
10	18:30-06:30	720	24	75	0

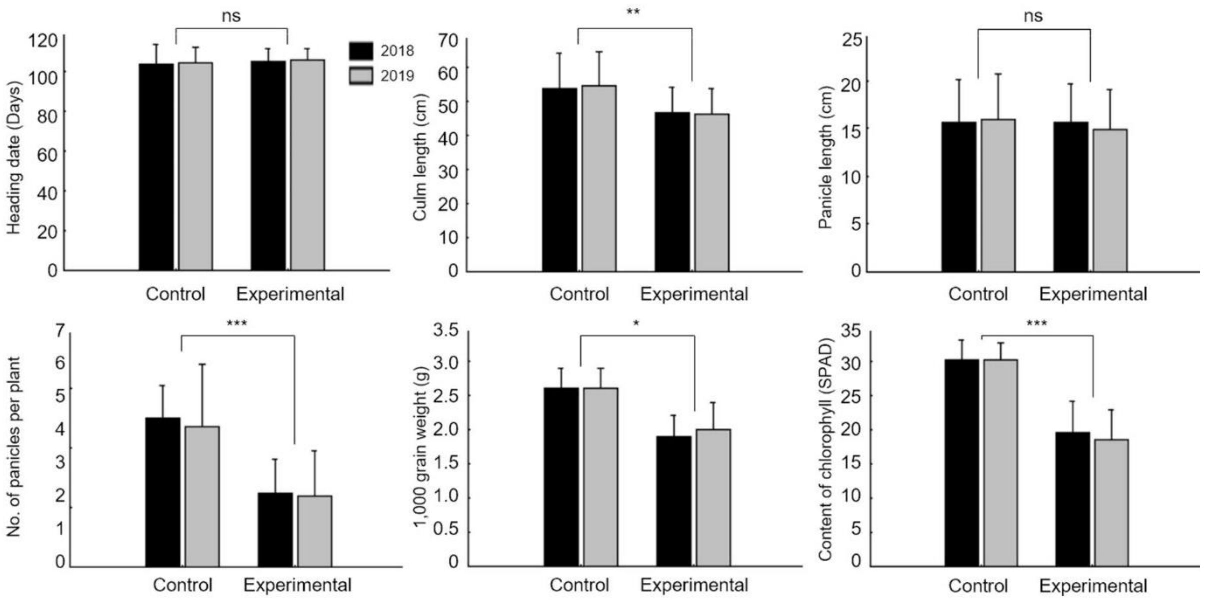

Figure S1. Comparison of agricultural traits of control and experimental groups when the rice in booting stage was treatment to high temperature. There was no significant difference in heading date and panicle length when booting stage was treated with high temperature. However, culm length (P <0.01), number of panicle (P <0.001), 1,000 grain weight (P <0.05), and content of chlorophyll (P <0.001) were significantly different between the control and experimental groups after high temperature treatment in booting stage. ns, non-specific; *, means significant at 0.05 level; **, means significant at 0.01 level; ***, means significant at 0.001 level.

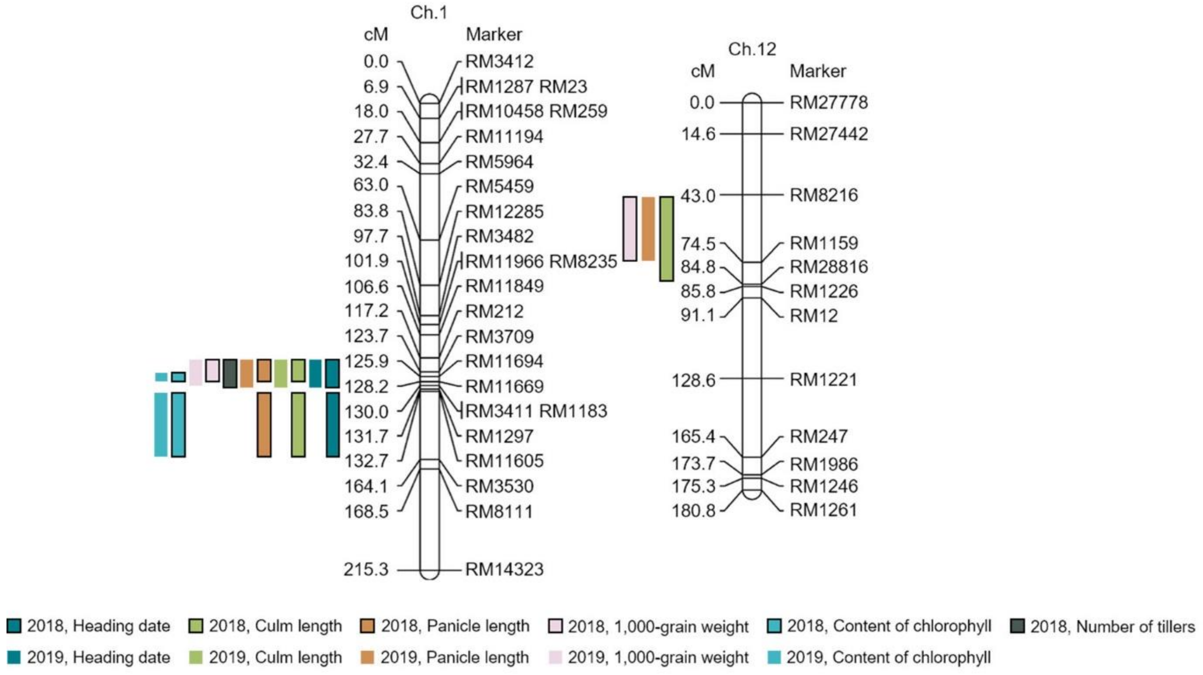

Figure S2. The chromosomal location of QTLs associated with heat tolerance in CNDH population.

The QTLs mapped in chromosome 1 and chromosome 12. And QTLs for all traits related to heat tolerance is commonly located in RM3709-RM11694 of chromosome 1.
